# Fluorescence Imaging of *Streptococcus pneumoniae* with the *Helix pomatia* agglutinin (HPA) As a Potential, Rapid Diagnostic Tool

**DOI:** 10.3389/fmicb.2017.01333

**Published:** 2017-07-18

**Authors:** Mirian Domenech, Ernesto García

**Affiliations:** ^1^Departamento de Microbiología Molecular y Biología de las Infecciones, Centro de Investigaciones Biológicas, Consejo Superior de Investigaciones Científicas Madrid, Spain; ^2^Centro de Investigación Biomédica en Red de Enfermedades Respiratorias Madrid, Spain

**Keywords:** *Streptococcus pneumoniae*, Forssman antigen, binding lectins, teichoic acids, fluorescence microscopy

## Abstract

*Streptococcus pneumoniae* is a common human pathogen and a major causal agent of life-threatening infections that can either be respiratory or non-respiratory. It is well known that the *Helix pomatia* (edible snail) agglutinin (HPA) lectin shows specificity for terminal αGalNAc residues present, among other locations, in the Forssman pentasaccharide (αGalNAc1→3βGalNAc1→3αGal1→4βGal1→4βGlc). Based on experiments involving choline-independent mutants and different growth conditions, we propose here that HPA recognizes the αGalNAc terminal residues of the cell wall teichoic and lipoteichoic acids of *S. pneumoniae*. In addition, experimental evidence showing that pneumococci can be specifically labeled with HPA when growing as planktonic cultures as well as in mixed biofilms of *S. pneumoniae* and *Haemophilus influenzae* has been obtained. It should be underlined that pneumococci were HPA-labeled despite of the presence of a capsule. Although some non-pneumococcal species also bind the agglutinin, HPA-binding combined with fluorescence microscopy constitutes a suitable tool for identifying *S. pneumoniae* and, if used in conjunction with Gram staining and/or other suitable technique like antigen detection, it may potentially facilitate a fast and accurate diagnosis of pneumococcal infections.

## Introduction

*Streptococcus pneumoniae*, the pneumococcus, is a leading human pathogen and one of the foremost etiologic agents of invasive diseases such as bacteremic community-acquired pneumonia (CAP), bacteremia, and meningitis, mainly in children, the elderly, and immunocompromised patients. Indeed, the pneumococcus is one of the major causes of non-invasive diseases such as non-bacteremic CAP, acute otitis media, sinusitis and conjunctivitis. In 2015, CAP accounted for 16% of all deaths of children under 5 years old globally and 920,000 deaths globally in children of all ages (World Health Organization, 2016). *S. pneumoniae* is the commonest bacterial causative agent across all ages, accounting for 30–40% of CAP cases (Haq et al., 2017). Other bacterial causes of CAP include *Streptococcus pyogenes* (group A streptococci; GAS), and *Streptococcus agalactiae* (group B streptococci; GBS) in infants. *Staphylococcus aureus* is associated with round pneumonia, a well-defined round area of consolidation visible on chest radiographs. Despite the current vaccination programs, *Haemophilus influenzae* remains prevalent in several developing countries (Hajjeh et al., 2013) and *Mycoplasma pneumoniae* accounts for up to a third of all cases and is a common cause of atypical CAP.

Since mortality rates are highest during the first days of admission (Ewig et al., 2009), early diagnosis and treatment may have a crucial role in curing the patient or in reducing their morbidity and mortality, particularly in the era of antibiotic resistance (Vila et al., 2017). Rapid identification techniques are even more important in bacterial meningitis patients, since delayed initiation of antibiotic treatment is strongly associated with death and poor outcome; as a consequence, it has been recently recommended that antibiotic treatment in these patients should be started as soon as possible, and the time period from entering the hospital to initiation of antibiotic treatment should not exceed 1 h (van de Beek et al., 2016). In every case, the gold standard diagnostic method is still culture. Unfortunately, this is time consuming (24–72 h) and includes the inoculation of appropriate media, subculturing, and phenotype-based characterization via biochemical testing, along with antibiotic susceptibility testing. Currently, bacteriological diagnosis is making progress in molecular biology techniques such as PCR, matrix-assisted laser desorption/ionization time-of-flight mass spectrometry (MALDI-TOF MS), and whole genome sequencing (Clark, 2015; Pai et al., 2015; McGill et al., 2016; Torres et al., 2016). These techniques have been developed to decrease the time for the initiation of an optimal antimicrobial therapy for improving clinical outcomes. Unfortunately, although microbiological diagnosis is very important for a correct clinical management of the disease—particularly because spreading of antibiotic multiresistance is an increasing global concern—, microbiological identification is lacking in many instances approaching 50% in CAP cases (Cilloniz et al., 2016).

Most bacteria in nature exist in multispecies communities known as biofilms. Multispecies biofilms are structured and spatially defined communities where species interact both intra- and interspecifically (Røder et al., 2016). Imaging techniques are useful for identifying multiple species, which provides information on their spatial organization. Fluorescence *in situ* hybridization (FISH) and many advanced versions of the FISH technique have been implemented for different purposes; these are well-established means of visualizing and identifying microbial groups or species in natural and artificially created multispecies biofilms (Amann and Fuchs, 2008; Liu et al., 2016; Røder et al., 2016). Although FISH is typically time-consuming and destructive to the samples, it is however possible to visualize different species simultaneously (Schimak et al., 2016). Fluorescence imaging of individual species can also be achieved by genetically marking the species with genes coding for different fluorescent or bioluminescent proteins (Kjos et al., 2015). Unfortunately, not all bacteria can be fluorescently tagged, particularly those from natural samples.

Polymicrobial biofilms are abundant in clinical diseases such as acute otitis media, a significant public health problem worldwide (Monasta et al., 2012), particularly among children (Ahmed et al., 2014). Acute otitis media is preceded by the nasopharyngeal carriage of bacterial pathogens like *S. pneumoniae*, non-typeable (NT) *H. influenzae*, and *Moraxella catarrhalis* (Ngo et al., 2016). Moreover, the concurrent carriage of these pathogens is a predictor of clinical pneumonia (Chochua et al., 2016). When growing in an *in vitro* mixed biofilm, *S. pneumoniae* and NT *H. influenzae* cells appear to accomplish a strong inter-population cooperation, i.e., metabolic interdependence or mutualism (Momeni et al., 2013), as indicated by the finding that pneumococci were intermixed with NT *H. influenzae* cells throughout the biofilm (Domenech and García, 2017). This was determined using the *Helix pomatia* (edible snail) agglutinin (HPA) that unambiguously stained *S. pneumoniae* cells in the mixed biofilms. The binding preference of HPA has been reported to be the Forssman pentasaccharide (αGalNAc1→3βGalNAc1→3αGal1→4βGal1→4βGlc) >blood group A substance (αGalNAc1→3[αFuc1→2]Gal] >Tn antigen (αGalNAc-Ser/Thr) >GalNAc >GlcNAc, confirming its specificity for terminal αGalNAc residues (Wu and Sugii, 1991; Cooling, 2015). The Forssman antigen (FA) can be defined as a substance that provokes the appearance of sheep red blood cell hemolytic antibodies when injected into rabbits; it is a glycolipid with the structure GalNAcα1→3GalNAcβ1→3Galα1→4Galβ1→4Glcβ1→1Cer (Siddiqui and Hakomori, 1971). Originally found in the tissues of different animals (although not humans; Yamamoto et al., 2012), FA was subsequently discovered in some bacteria, including *S. pneumoniae* (Jenkin, 1963). It has been shown that the Forssman cross-reactive material of *S. pneumoniae* is the type IV, ribitol phosphate-containing, membrane anchored lipoteichoic acid (pnLTA) (Briles and Tomasz, 1973; Gisch et al., 2013). The non-lipid terminus of pnLTA consists of 6-*O*-*P*Cho-α-D-Gal*p*NAc-(1→3)-6-*O*-*P*Cho-β-D-Gal*p*NAc (Seo et al., 2008); this disaccharide represents a structural feature that is able to partly explain the FA properties of pnLTA (Gisch et al., 2013). In addition and unlike other bacterial species, pnLTA and the *S. pneumoniae* peptidoglycan-bound teichoic acid (pnWTA) have identical chain structures (for a recent review, see reference by Gisch et al. (2015a). Based on this information it can be assumed that HPA binds to both WTA and LTA of *S. pneumoniae*.

In the present study we report that HPA binds to the teichoic acids (TA) of encapsulated and non-encapsulated pneumococcal cells grown either planktonically or forming biofilms. In addition to *S. pneumoniae*, HPA labeling of other bacterial species, e.g., some *S. aureus* strains, has also been observed. We propose that, in combination with other widespread rapid techniques, labeling with HPA in biological fluids may represent a helpful technique for the fast and accurate diagnosis of pneumococcal diseases.

## Materials and methods

### Bacteria and growth conditions

The bacteria used in this study are listed in Table 1. Streptococci, staphylococci, enterococci, and *Pseudomonas aeruginosa* were grown in Todd-Hewitt broth supplemented with 0.5% yeast extract (THY). For planktonic growth, the NT *H. influenzae* strain 54997 was incubated in brain heart infusion (BHI) supplemented with haemin and NAD (15 μg/ml each) (sBHI). In some experiments, *S. pneumoniae* was grown in C medium (Lacks and Hotchkiss, 1960) supplemented with 0.08% yeast extract (Difco Laboratories; C+Y medium) or a chemically defined medium (Cden) supplemented (or not) with either choline (5 μg/ml) (Cden-choline) or ethanolamine (40 μg/ml) (Cden-EA) (Tomasz, 1968). Cells were incubated at 37°C without shaking. Bacterial growth was monitored by measuring the absorbance at 595 nm (*A*_595_).

**Table 1:** Bacterial strains used in this study.

**Strain**	**Description**	**Source or References^a^**	**HPA labeling^b^**
*S. pneumoniae*			
D39	Serotype 2; clinical isolate	Lanie et al., 2007	+
P007	Serotype 3; laboratory transformant	Domenech et al., 2009	+
R6	Non-encapsulated D39 derivative	Hoskins et al., 2001	+
MNZ67	Non-encapsulated; clinical isolate	Park et al., 2012	+
1001	Serotype 3; clinical isolate	J. Liñares^c^	+
2951/13	Serotype 12F; clinical isolate	Domenech et al., 2015	+
Tupelo_VT	Serotype 14; clinical isolate	Moscoso et al., 2010	+
1064	Serotype 19F; clinical isolate	A. Fenoll^d^	+
1178/17	Serotype 22F; clinical isolate	L. Vicioso^d^	+
2971/13	Serotype 23B; clinical isolate	Domenech et al., 2015	+
JY2190	Choline-independent Rx1 mutant; non-encapsulated	Yother et al., 1998	+
P501	Choline-independent R6 mutant; non-encapsulated	González et al., 2008	+
*Streptococcus pseudopneumoniae*			
CCUG 49455	Type strain	CCUG	–
*Streptococcus mitis*			
NCTC 12261	Type strain	NCTC	–
SK137	Biovar 1; strain with choline-containing teichoic acids	Bergström et al., 2000	+
SK598	Biovar 1; strain with ethanolamine-containing teichoic acids	Bergström et al., 2003	–
*Streptococcus oralis*			
NCTC 11427	Type strain	Kilpper-Bälz et al., 1985	–
*Streptococcus pyogenes*			
CECT 985	Type strain; Lancefield group A	CECT	–
*Streptococcus agalactiae*			
CECT 183	Type strain; Lancefield group B	CECT	–
*Streptococcus dysgalactiae* subsp. *equisimilis*			
CECT 926	ATCC 9542; Lancefield group C	CECT	+
*Streptococcus suis*			
298	Serotype 9; Lancefield group D^e^	A. I. Vela^f^	+/–
357	Serotype 2; Lancefield group D	A. I. Vela	+/–
*Staphylococcus aureus*			
CECT 86	Type strain	Shiroma et al., 2015; CECT	+
15981	Clinical isolate; good biofilm former	Valle et al., 2003	+
*Staphylococcus epidermidis*			
CECT 231	Good biofilm former	Domenech et al., 2011; CECT	–
*Enterococcus faecalis*			
CECT 481	Type strain	CECT	–
*Haemophilus influenzae*			
54997	Nontypeable	García-Cobos et al., 2014	–
*Pseudomonas aeruginosa*			
PAO1	ATCC 15692	ATCC	–

a *ATCC, American Type Culture Collection; CECT, Colección Española de Cultivos Tipo; CCUG, Culture Collection, University of Göteborg; NCTC, National Collection of Type Cultures*.

b *+, positive; −, negative; +/−, slightly positive*.

c *Instituto de Investigación Biomédica de Bellvitge (IDIBEL); Barcelona (Spain)*.

d *Laboratorio de Referencia de Neumococos; Centro Nacional de Microbiología (CNM-ISCIII); Majadahonda (Madrid; Spain)*.

e *Although early studies reported that some strains of S. suis contained streptococcal group D antigen, more recent results indicated that the group R and group D antigens were similar and crossreacted. To date, the species belonging to the Streptococcus bovis group constitute the non-enterococcal group D streptococci (Dekker and Lau, 2016)*.

f *Facultad de Veterinaria; Universidad Complutense de Madrid; Madrid (Spain)*.

Formation of mixed biofilms of *S. pneumoniae* and NT *H. influenzae* was performed as described elsewhere (Domenech and García, 2017). Briefly, cultures of *S. pneumoniae* strain R6 and *H. influenzae* strain 54997 were grown to mid-exponential phase in C+Y medium supplemented with haemin and NAD (15 μg/ml each) [s(C+Y)], diluted to ≈5 × 10^6^ colony-forming units (cfu)/ml and mixed in an 1:1 proportion. Two milliliter of the mixtures were then distributed into the wells of a glass-bottomed dish (WillCo-dish, WillCo Wells B. V., The Netherlands) and incubated for 6 h at 37°C under 5% CO_2_. For species biofilm formation, 2 ml of the individual cultures (5 × 10^6^ cfu/ml each) were independently inoculated as indicated above for mixed biofilms.

All studies which involved the handling of virulent bacteria, whole blood, or blood derivatives were undertaken at the biosafety level II laboratory of Centro de Investigaciones Biológicas. It should be mentioned that, according to the supplier (Innovative Research), the whole human blood used had been tested by FDA-approved methods for human immunodeficiency virus RNA, antibodies to immunodeficiency virus, antibodies to hepatitis C virus, hepatitis C virus RNA, hepatitis B virus, hepatitis B surface antigen, and syphilis.

### Staining with HPA lectin

Exponentially growing cells of various bacterial species were centrifuged, washed and suspended in phosphate-buffered saline (PBS). After incubation for 15 min at room temperature in the dark with HPA lectin conjugated to Alexa Fluor-488 (2.5–25 μg/ml), cells were centrifuged again and resuspended in PBS. Bacteria were observed under a Leica DM4000B fluorescence microscope equipped with an L5 filter (bandpass 480/40), and viewed under a Leica HCX PL FLUOTAR 40×/0.75 objective or an HC PL APO 63×/1.40–0.60 oil objective. In early experiments, bacteria were also diluted into fetal bovine serum (from Sigma-Aldrich), or into defibrinated sheep blood (from Biomedics or Oxoid). Afterwards, experiments were also carried out with groups A and O citrated human whole blood (from Innovative Research). It should be underlined that since HPA labeling does not require the presence of divalent cations (Kobayashi et al., 2014), blood treated to prevent its coagulation (e.g., citrate- or EDTA-treated) can be used.

For biofilm observation, the culture medium was removed and the biofilm rinsed with sterile water to remove non-adherent bacteria. Staining was performed with HPA and SYTO 59 and biofilms were gently rinsed with PBS. Observations were made using a Leica TCS-SP2-AOBS-UV confocal laser scanning microscope (CLSM) equipped with an argon ion laser. Images were analyzed using LCS software from Leica. Projections were obtained in the planes *x*–*y* (individual scans at 0.5 μm intervals) and *x*–*z* (images at 5 μm intervals).

## Results

### HPA binding by different bacteria

**Figure 1:**
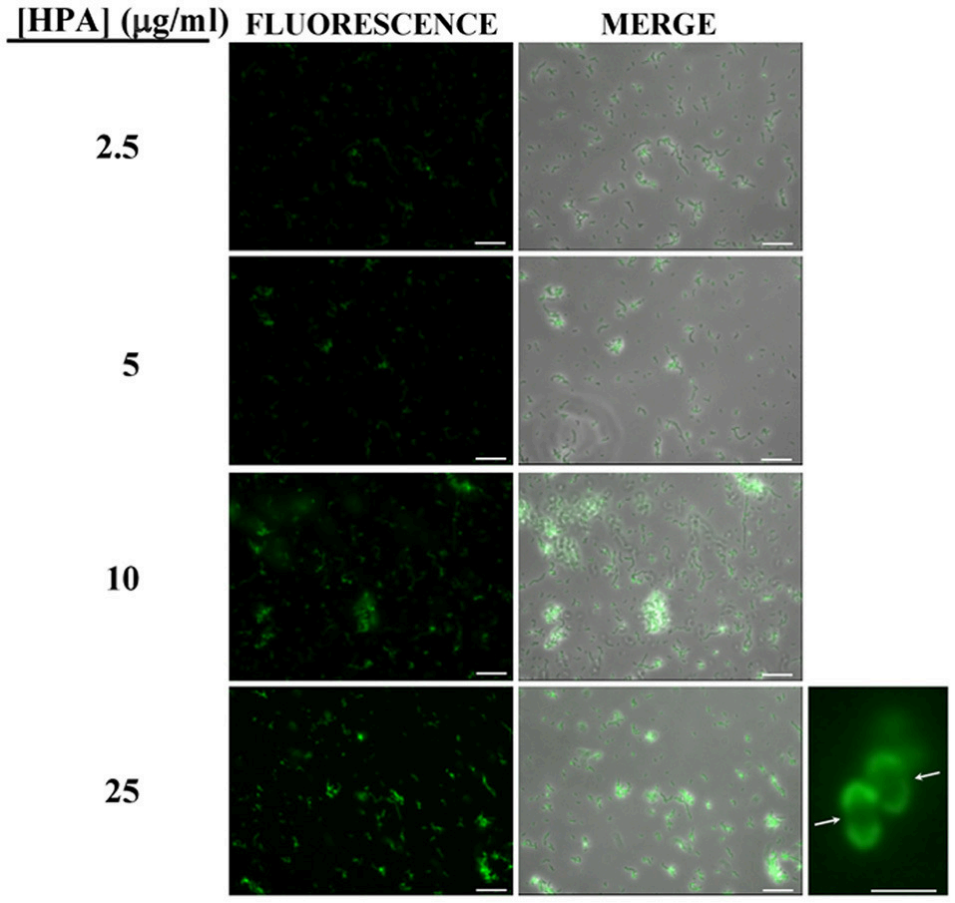
Fluorescent labeling of the non-encapsulated *S. pneumoniae* strain R6 with HPA. Exponentially growing cultures of *S. pneumoniae* R6 in C+Y medium were incubated with the indicated concentrations of the lectin and observed for fluorescence (HCX PL FLUOTAR 40×/0.75 objective). Merges of fluorescence and phase-contrast images are also shown; bar = 25 μm. Enlarged view of two diplococci showing reduced fluorescence at the equatorial zone of growth (indicated by arrows; 63× objective). Bar = 2 μm.

**Figure 2:**
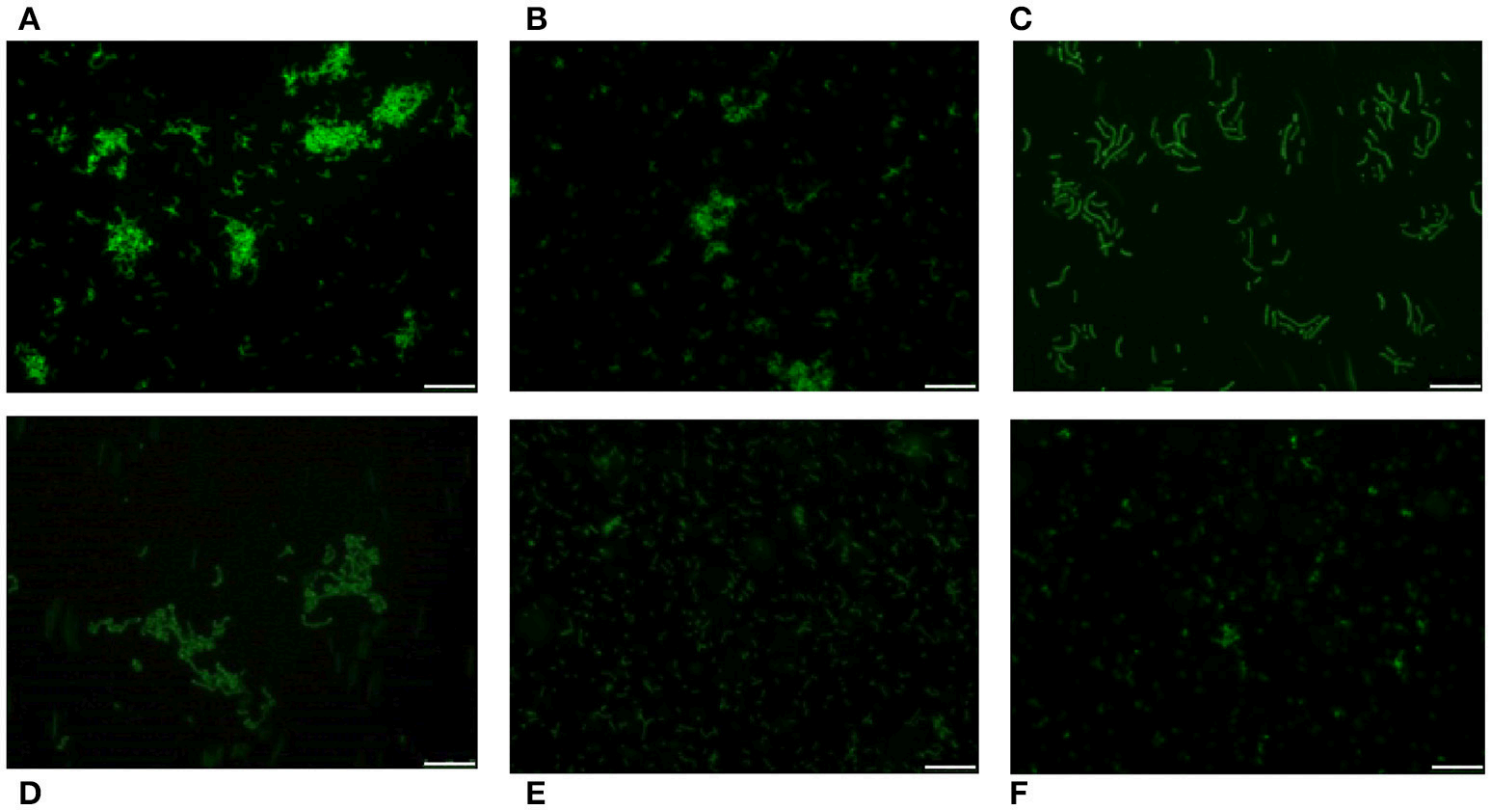
HPA labeling of various streptococci in C+Y medium. First row: *S. pneumoniae* strains R6 (non-encapsulated) **(A)**, D39 (serotype 2) **(B)**, and P007 (serotype 3) **(C)**. Second row: *S. suis* 298 **(D)**, *S. dysgalactiae* subsp. *equisimilis* CECT 926 **(E)**, and *S. aureus* type strain **(F)**. Bar = 25 μm.

**Figure 3:**
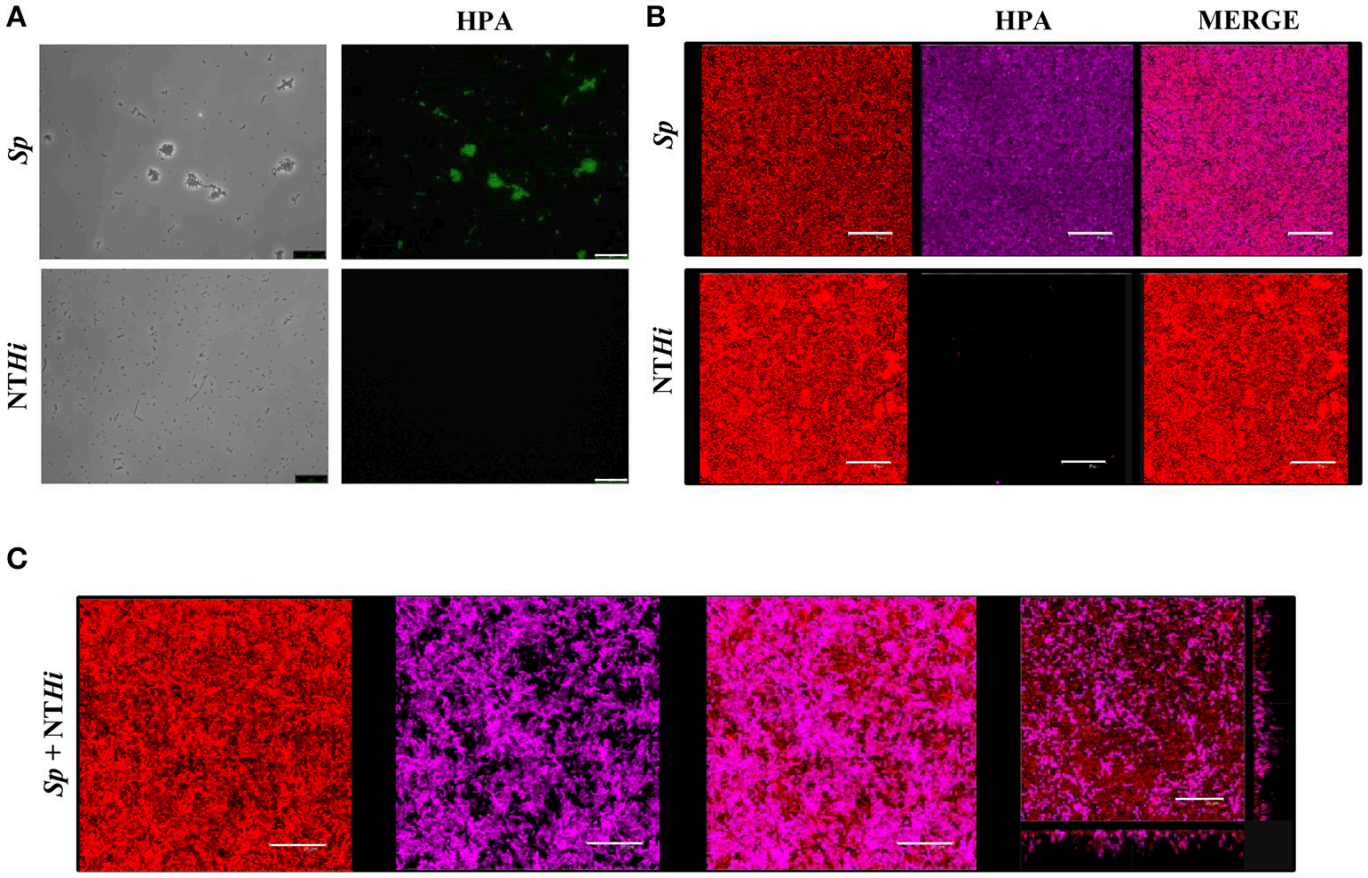
*S. pneumoniae* labeling with HPA in mixed biofilms. **(A)** Fluorescent labeling of the *S. pneumoniae* (*Sp*) strain R6 and the non-typeable *H. influenzae* (NT*Hi*) 54997 with HPA. Biofilms formed by *Sp* R6 or NT*Hi* 54997 **(B)**, or both pathogens **(C)** were stained with a combination of SYTO 59 (red) and HPA conjugated to Alexa Fluor-488 (pink). An orthogonal projection of a CLSM image showing a representative region of the *x–y* plane over the depth of the biofilm in both *x–z* and *y–z* dimensions of the mixed biofilm is also shown at the bottom right part of the figure. Planktonic and biofilms cultures were incubated with HPA (25 μg/ml). Bar = 25 μm.

Pneumococci were clearly labeled with HPA when this was present in concentrations of 2.5–25 μg/ml (Figure 1). Closer examination showed that HPA labeling was not uniform across the pneumococcal surface; reduced fluorescence was noticed in the equatorial zone of growth, the place where new cell wall material is incorporated (Gisch et al., 2015a). The ability to bind HPA was not exclusive to pneumococci; the surfaces of other bacteria too were labeled with the lectin, i.e., *Streptococcus mitis* SK137 and some strains of *Streptococcus dysgalactiae* subsp. *equisimilis, Streptococcus suis* and *S. aureus* (Figure 2, Table 1). Notably, HPA was unable to label the type strains (^T^) of *Streptococcus pseudopneumoniae, S. mitis*, or *Streptococcus oralis*, the three closest relatives of the pneumococcus. Notably, cells of other relevant pathogens, either Gram-positive (GAS, GBS, *Enterococcus faecalis* and *Staphylococcus epidermidis*) or Gram-negative (*H. influenzae* and *P. aeruginosa*), did not bind the lectin (Table 1). Besides, and in agreement with previous results (Domenech and García, 2017), *S. pneumoniae* (but not NT *H. influenzae*) were also labeled with HPA when growing as mixed biofilms (Figure 3).

**Figure 4:**
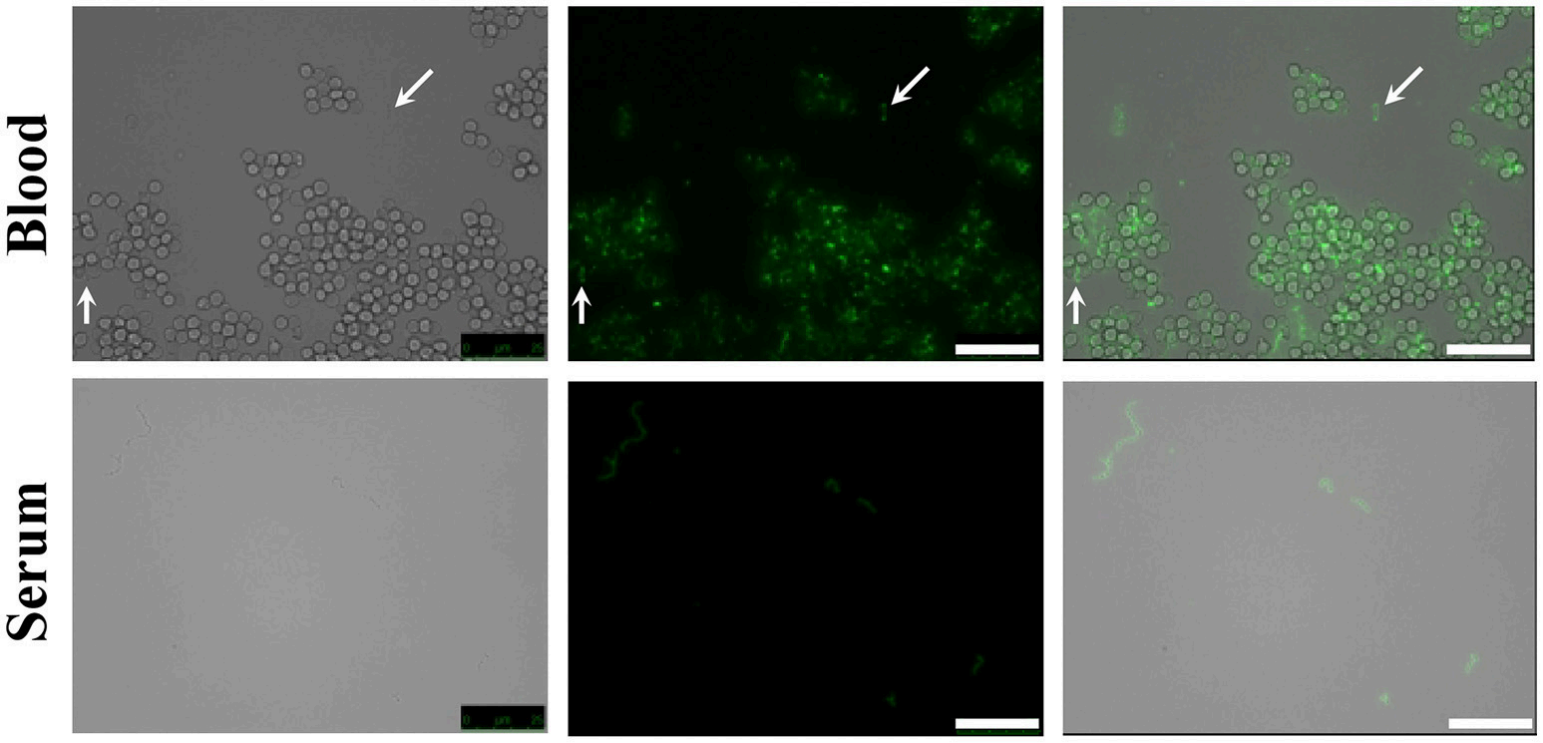
HPA labeling of *S. pneumoniae* in non-human blood and serum. The type 2 encapsulated *S. pneumoniae* strain D39 was diluted to 2.5 × 10^6^ cfu/ml into sheep blood or fetal bovine serum (63× objective). Cultures were incubated with HPA (25 μg/ml). Arrows point to pneumococcal cells. Bar = 25 μm.

**Figure 5:**
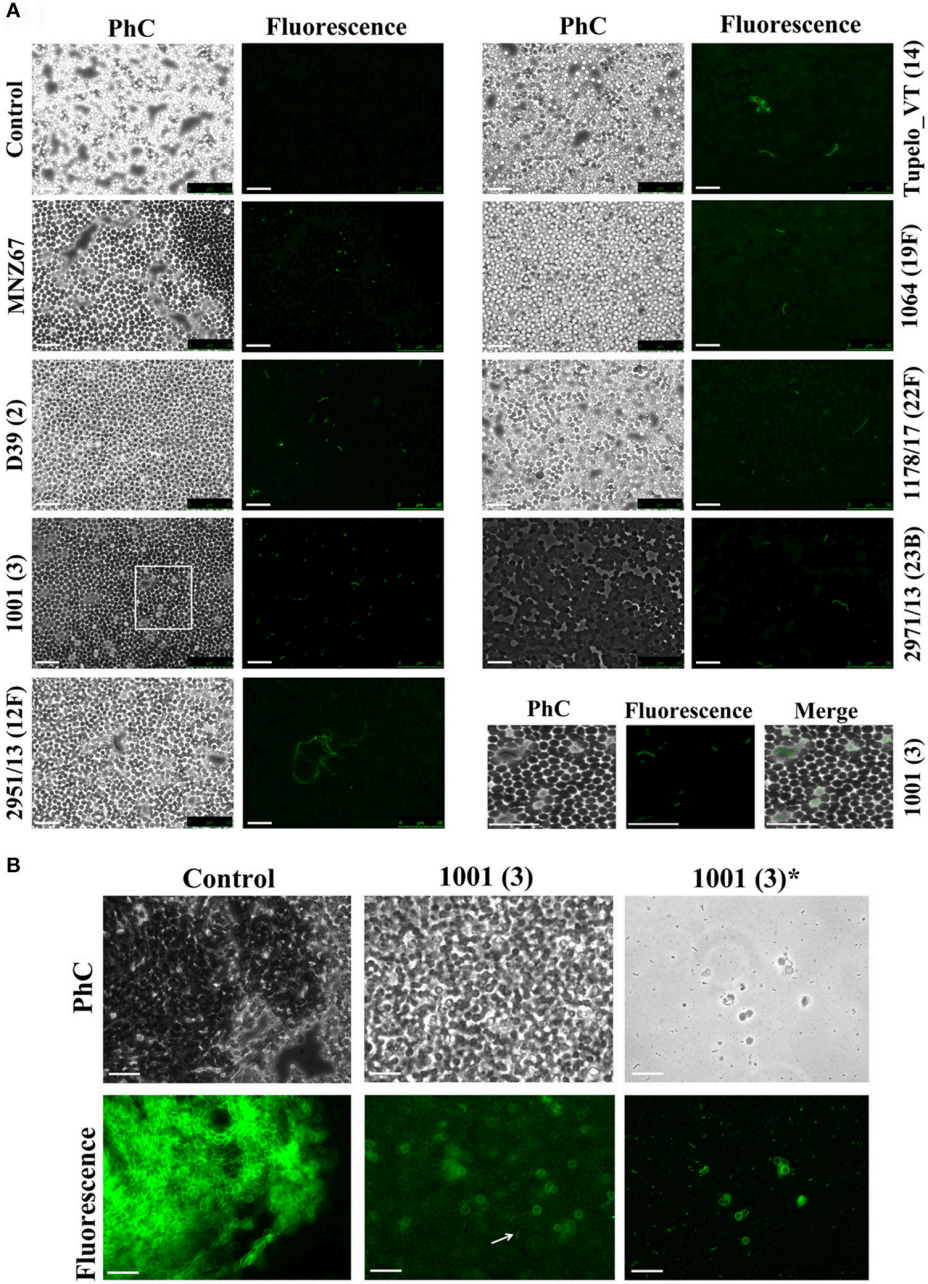
HPA labeling of clinical pneumococcal isolates in human blood. The *S. pneumoniae* strains were diluted to 2.5 × 10^6^ cfu/ml into group O **(A)** and A **(B)** human blood (40× objective). Serotypes are indicated in parentheses. Strain MNZ67 is a non-encapsulated clinical isolate. In **(A)**, the images shown at the bottom right are enlarged visions of the area marked with a rectangle in strain 1001. Control denotes non-infected blood samples. In **(B)**, those labeled with an asterisk correspond to views of the supernatant of infected group A blood that had been centrifuged (1,000 × g; 1 min; room temperature) before HPA labeling. Samples were incubated with HPA (25 μg/ml). The arrow points to a pneumococcal diplococcus. Phc, phase-contrast micrograph; Bar = 25 μm.

The presence of the capsule does not appear to hinder HPA binding to the *S. pneumoniae* surface since strains D39 (serotype 2) and P007 (a heavily encapsulated serotype 3 transformant) were efficiently labeled (Figures 2B,C). Cells of *S. pneumoniae* D39 were also positive for HPA binding when diluted to 2.5 × 10^6^ cfu/ml into defibrinated sheep blood or into fetal bovine serum, although excess fluorescence on the erythrocyte surface may partly hinder a distinct pneumococcal identification (Figure 4). As HPA also binds to the human blood group A antigen (Matsui et al., 2001), that interference, which is due to the fact that sheep erythrocytes harbor FA on their surface (see above), should disappear using group non-A (e.g., group O) human whole blood. In addition, it should be mentioned that up to 98 pneumococcal capsular polysaccharides differing in sugar composition and linkages have been described to date (Geno et al., 2017). Besides, invasive pneumococcal disease and pneumonia rates have decreased in most countries following the introduction of conjugate pneumococcal vaccines (PCVs). However, after PCV implementation, current data show that more non-vaccine serotypes increased in frequency than decreased, which is consistent with vaccine-induced replacement. Clinical pneumococcal isolates of six different serotypes—including three emerging serotypes that are currently among the predominant non-PCV13 serotypes worldwide, i.e., serotypes 12F, 22F, and 23B; Balsells et al., 2017)—were diluted to about 2.5 × 10^6^ cfu/ml into whole human blood, incubated with HPA, and observed under the microscope. Moreover, due to the increasing clinical importance of non-encapsulated *S. pneumoniae* (Keller et al., 2016), a representative strain (MNZ67) was also investigated. All the pneumococcal isolates tested bound the lectin and, as expected, HPA-labeled pneumococci were particularly noticeable using group O human blood (Figure 5). It is worth mentioning that any possible interference caused by an excess fluorescence on the surface of group A human erythrocytes could be virtually abolished by partly sedimenting the blood cells at low speed (1,000 × g; 1 min; room temperature) before HPA addition (Figure 5A, bottom right).

### HPA labeling of pneumococcal teichoic acids

**Figure 6:**
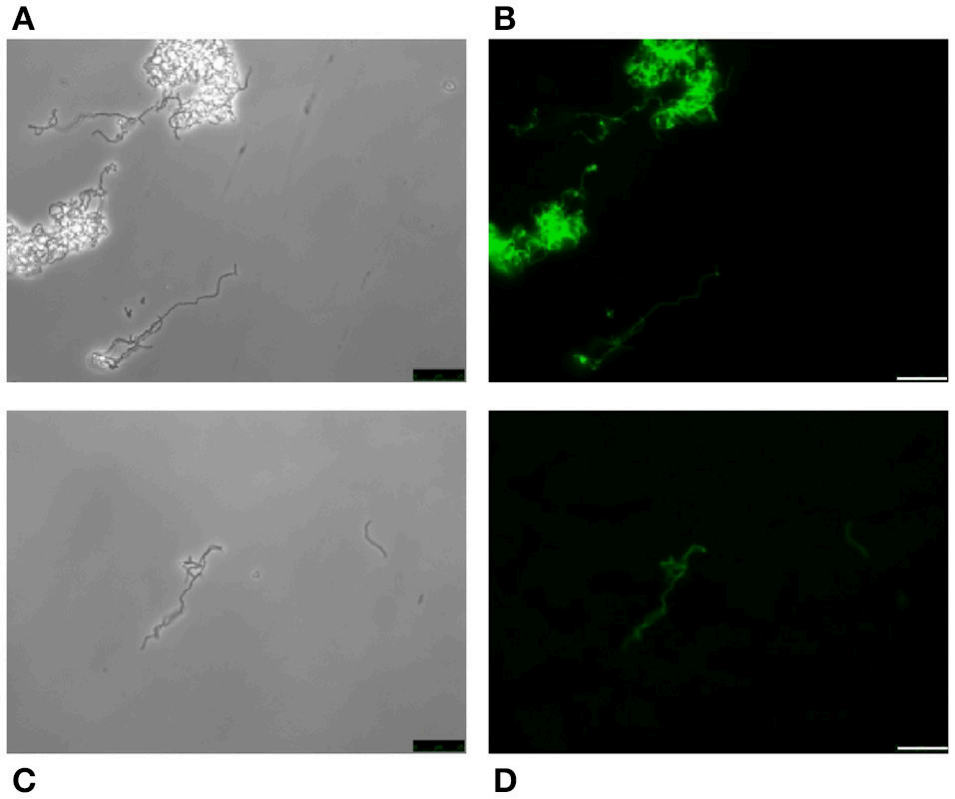
HPA labeling of two choline-independent *S. pneumoniae* strains. Pneumococcal strains JY2190 **(A,B)** and P501 **(C,D)** were incubated in a chemically-defined medium (Cden) lacking any amino alcohol, and labeled with HPA (25 μg/ml). Bar = 25 μm.

With the possible exception of *S. pseudopneumoniae* (González et al., 2008), the nutritional requirement of pneumococci for the amino alcohol choline (as a component of pnWTA and pnLTA) appears to be an exclusive trait (Rane and Subbarow, 1940). Nevertheless, several choline-independent pneumococcal mutants have been characterized in the last years, and it has been suggested that the absence of choline incorporation might affect the structure of TAs as well as the composition of the cell wall. Actually, it has been shown that pnLTA and pnWTA isolated from these mutants were free of phosphocholine and other phosphorylated aminoalcohols (Yother et al., 1998). However, and as deduced from the *in vivo* cell labeling with HPA of two double *tacF* mutants that form long chains of cells (and are autolysis-defective) when grown in media lacking any amino alcohol (strains JY2190 and P501), the absence of choline residues in TAs does not appear to modify HPA binding by *S. pneumoniae* (Figure 6).

**Figure 7:**
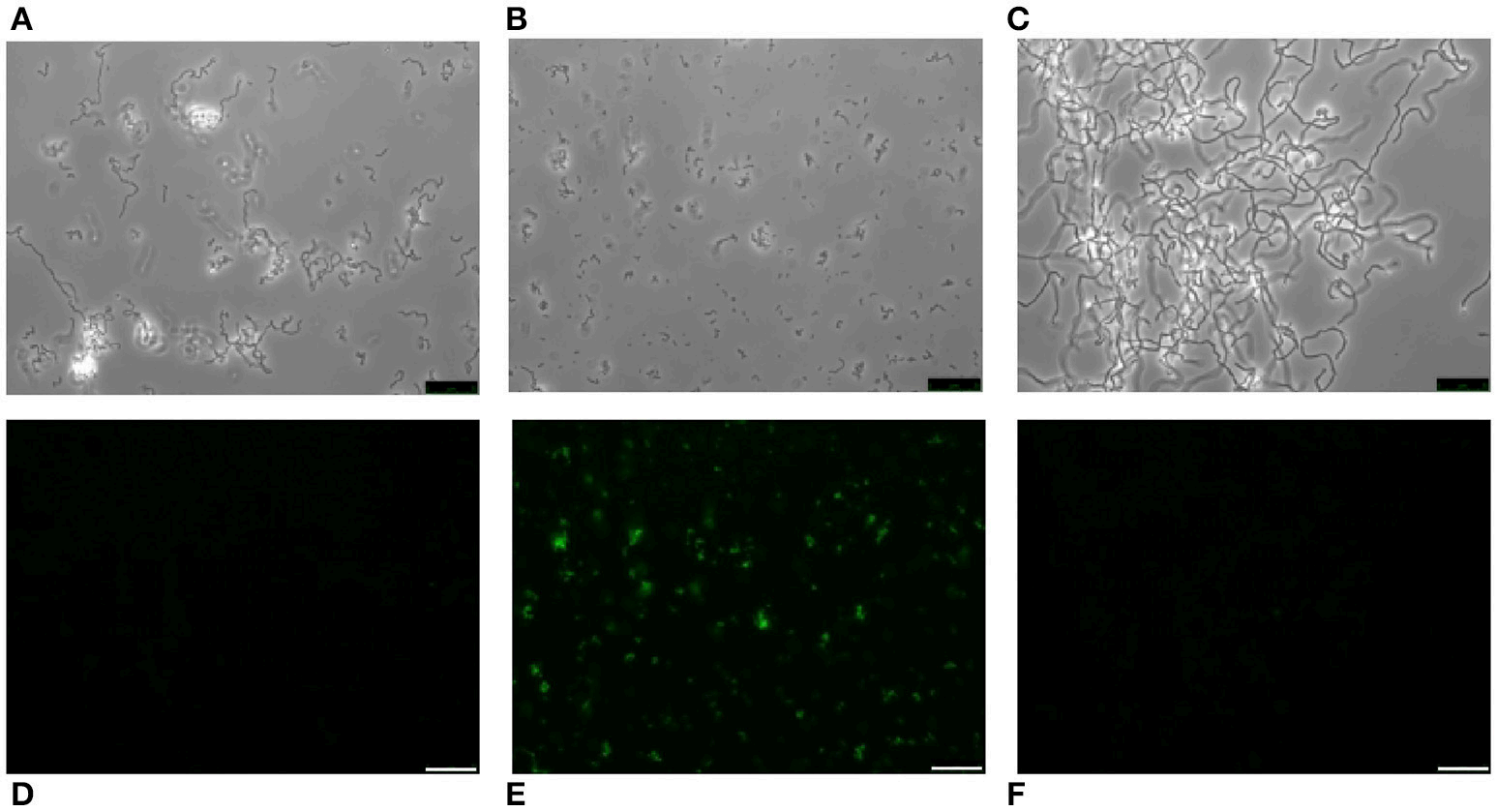
Lack of HPA labeling in ethanolamine-containing streptococci. *S. pneumoniae* R6 cells were incubated for several generations in Cden medium containing ethanolamine (Cden-EA) **(A,D)**. A portion of the culture then received choline chloride (5 μg/ml) and incubation proceeded at 37°C for 3 h **(B,E)**. The *S. mitis* SK598 strain was grown in THY medium **(C,F)**. The three cultures were incubated with HPA (25 μg/ml) **(D–F)**. Bar = 25 μm.

It is well known that pneumococci growing in Cden medium containing EA instead of choline form long chains, do not autolyze, and are non-transformable (Tomasz, 1968). In the present work, the EA-grown cells were unable to bind HPA (Figures 7A,D). The same was seen when *S. pneumoniae* R6 was incubated in C+Y supplemented with 2% choline chloride (data not shown). It has been reported that upon the addition of choline (5 μg/ml) to EA-grown pneumococci, these cells revert to the normal phenotype (Tomasz et al., 1975). As expected, EA-grown cells became entirely HPA-labeled 180 min (≈4 generations) after shifting to a choline-containing medium (Figures 7B,E). As an alternative and complementary model, we examined the *S. mitis* strain SK598, which is unique in that its WTA and LTA contain EA instead of choline even when incubated in a choline-containing medium (Bergström et al., 2003). As observed for EA-grown *S. pneumoniae* cells, *S. mitis* SK598 was unable to bind HPA (Figures 7C,F).

## Discussion

In a previous work we have shown that, in contrast to *S. pneumoniae*, NT *H. influenzae* strains did not bind HPA; thus fluorescent staining of *S. pneumoniae* with the HPA lectin revealed that pneumococci were evenly distributed throughout the *in vitro* biofilm and interspersed with NT *H. influenzae* (Domenech and García, 2017). We have shown here that HPA recognition of the *S. pneumoniae* surface does not require the presence of choline residues in TAs. This observation is in agreement with the results of Gisch et al. who recently reported that the presence/absence of phosphorylcholine in the FA terminus of pnLTA to have no effect on detection by an anti-Forssman antibody (Gisch et al., 2013). This is relevant since the number of repeating units and the phosphocholine content per repeat (mono-substituted or di-substituted) for pneumococcal TAs slightly vary among strains (Gisch et al., 2015a). It is interesting, however, that, in the present work, EA-grown cells or *S. pneumoniae* R6 incubated in C+Y supplemented with 2% choline chloride did not bind HPA. Incubation in high choline concentrations inhibits cell separation, leaving pneumococci to grow in long chains of cells, just like EA-grown cells (Briese and Hakenbeck, 1984; Giudicelli and Tomasz, 1984). This phenotype is, at least partly, the result of the inhibition of LytB—a member of the family of choline-binding proteins (CBPs)—and the release of this and other CBPs to the medium (López and García, 2004). Both processes would be expected to occur in choline-independent strains growing in the absence of any amino alcohol (see above), although when this was performed in the present work HPA-labeling was unaffected. Interestingly, in an early study, Briles and Tomasz reported the yield of heterophile (sheep hemolytic) antibodies elicited by pneumococci to be at least 10-fold greater in choline- than in EA-containing media (Briles and Tomasz, 1975). Moreover, these authors reported that pneumococci growing in C+Y medium (choline-containing) elicit antibodies which bind poorly to EA-grown bacteria, whereas the latter elicit antibodies which bind well to choline-grown cells. The reasons for these unexpected results are still unclear, although the possibility of the existence of a choline-dependent regulatory pathway for the synthesis of pneumococcal TAs warrants future research.

To our knowledge, the concentration of free choline (or EA) in human lungs has not been reported so far. However, the normal levels of free choline and EA in human fluids are quite similar, e.g., about 1 μM each in serum (Forteschi et al., 2016; Dereziński et al., 2017) and 2 μM for choline or 9–15 μM for EA in cerebrospinal fluid (CSF) (Kruse et al., 1985; Frölich et al., 1998; Ogawa et al., 2015). These data together with the early findings that choline is an effective inhibitor of the cellular incorporation of EA: addition of as little as 0.1 μg/ml choline to a culture growing in the presence of 40 μg/ml EA immediately inhibited further EA incorporation, and that choline was incorporated by such cultures without any detectable lag (Tomasz, 1968), strongly suggest that EA-grown cells (or pneumococci grown in high choline concentrations) are not expected to be found in nature, although they represent important model systems for *in vitro* studies.

HPA labeling allows the recognition of *S. pneumoniae* cells among a variety of other species. However, it is clear that HPA specificity is not restricted to pneumococci (Table 1). Interestingly, neither *S. pseudopneumoniae*^T^, *S. mitis*^T^ nor *S. oralis*^T^ binds the lectin; this is of note since, although these three species are very closely related to *S. pneumoniae*, monoclonal antibodies directed against the backbone and the phosphocholine residues of TAs react only with some strains of these three species (Kilian et al., 2008). The binding of HPA to these bacteria could, therefore, be mostly strain-specific. For example, in contrast to that observed for *S. mitis*^T^, *S. mitis* SK137 was susceptible to HPA labeling (Table 1). This was not unexpected since this particular strain has choline-containing TAs with a carbohydrate backbone identical to that of pnWTA/pnLTA, which forms the Lancefield group O antigen (Bergström et al., 2000). Quite unexpectedly, the SK137 strain only showed an average 67.1% nucleotide similarity to the *S. mitis*^T^ in a DNA–DNA hybridization assay (Kilian et al., 2008), slightly below the 70% level typically expected for two strains of the same species (Wayne et al., 1987). Whether strain SK137 represents a distinct species is, however, debatable, according to recent taxonomic proposals (Tindall et al., 2010).

The type strain of *S. oralis* (NCTC 11427)—the LTA structure of which is unknown—did not bind HPA (Table 1). However, a recent study has revealed that, in contrast to pnLTA, in which the structural element αGalNAc1→3βGalNAc1→is present (Gisch et al., 2013), only a βGalNAc1→moiety is detectable in the *S. oralis* Uo5 LTA repeating unit (Gisch et al., 2015b). Assuming an identical LTA structure for *S. oralis*^T^, the lack of the αGalNAc1→residue at the non-reducing end would fit in with the absence of HPA labeling. It should be underlined that at least three biochemical variants of choline-containing TAs may occur in *S. oralis* and some *S. mitis* strains, according to recent results (Denapaite et al., 2016).

Among the few other bacterial species tested, Lancefield group C and D streptococci were positive for HPA labeling. These results were expected in view of previous reports of *Streptococcus* belonging to groups C (Coligan et al., 1977; Sørensen and Henrichsen, 1987; Köhler and Nagai, 1989) and D (Kurl et al., 1989). From a diagnostic perspective, however, it is important to underline that non-pneumococcal, viridans (i.e., α-hemolytic) streptococci are usually considered as commensals and seldom cause CAP. It is well known that viridans streptococci produce a range of invasive disease in humans (e.g., infective endocarditis) and are also emerging as a cause of bloodstream infections, but mainly in immunocompromised patients (Doern and Burnham, 2010). *S. dysgalactiae* subsp. *equisimilis*, which belongs to the β-hemolytic group C and G pyogenic group of streptococci, also binds HPA and is also currently considered as an emergent human pathogen. Nevertheless, and as mentioned above for viridans streptococci, it is a frequent cause of invasive disease only in patients having underlying conditions (Broyles et al., 2009).

*S. aureus* is an important opportunistic pathogen that persistently colonizes about 20% of the human population and is intermittently associated with the remainder. This organism is one of the most frequent and important human pathogens and is implicated in a range of infections, including superficial skin infections, abscesses, and food poisoning as well as life-threatening invasive diseases (Tong et al., 2015). Although, *S. aureus* possesses surface carbohydrates that might be recognized by HPA (Krivan et al., 1988; Payne et al., 1992), the molecular basis for HPA binding is not well understood. It has been shown, however, that certain methicillin-susceptible *S. aureus* isolates—in particular those belonging to the atypical sequence type (ST) 395 lineage (e.g., strain PS187; Winstel et al., 2013)—produce a glycerol TA modified with αGalNAc (Winstel et al., 2014; Lee et al., 2015) that may be responsible for HPA binding. It should be noted, however, that the chemical structures of WTA and LTA of *S. aureus* are similar but not identical (Xia et al., 2010; Brown et al., 2013), that the ST of the type strain of *S. aureus* (ST8) is only distantly related to ST395, and that strain PS187 appears to be more closely related to several coagulase-negative staphylococcal species than to other *S aureus* isolates (Winstel et al., 2013); the ST of *S. aureus* 15981 is unknown. It has been demonstrated that staphylococci can be accurately differentiated from streptococci in Gram-stained preparations (cocci in clusters and diplococci or short chains, respectively; Agger and Maki, 1978), and that *S. aureus* is more uncommon than the latter in CAP and acute meningitis cases. However, our results warrant additional studies to determine whether HPA labeling is common among *S. aureus* isolates, particularly in methicillin-resistant isolates that represent a global health care problem (Tong et al., 2015).

In agreement with a previous report (Domenech and García, 2017), NT *H. influenzae* strains did not bind HPA; identical results are shown here for *P. aeruginosa* PAO1. Together with *S. pneumoniae*, both Gram-negative species are common in biofilms formed during acute otitis media and live-threatening, chronic respiratory diseases such as chronic obstructive pulmonary disease or cystic fibrosis (Blasi et al., 2016). The use of HPA as *S. pneumoniae-*specific lectin for fluorescence imaging should provide a powerful tool for future research on these and other relevant human pathogens forming multispecies biofilms.

HPA labeling combined with Gram staining and/or antigen detection may also constitute an appropriate combination for the rapid diagnosis of CAP and, perhaps, other conditions such as bacterial meningitis (McGill et al., 2016). This may facilitate the rapid implementation of an appropriate antibiotic regime, which is conditional on the age of the patient and the regional rate of decreased susceptibility of *S. pneumoniae* to β-lactam antibiotics (van de Beek et al., 2016). As a proof of concept, cultures of pneumococci, *H. influenzae* and GBS were mixed either with sheep blood or fetal bovine serum. Also using human whole blood, only pneumococci (either encapsulated or non-encapsulated) were clearly identified by HPA labeling when used at a relatively low bacterial concentration (2.5 × 10^6^ cfu/ml). This concentration is lower than that frequently found in the blood of CAP patients (between 7 × 10^7^ and 8 × 10^8^ cfu/ml; Gadsby et al., 2016) or in the CSF of children with confirmed pneumococcal meningitis (median bacterial load ≈5 × 10^7^ DNA copies/ml) at the time of admittance (Roine et al., 2009). If required, bacteria could be concentrated by centrifugation before or after staining.

There are a number of limitations to our study: (1) although the HPA labeling technique does not require a very specialized personnel, fluorescence microscopy may be unavailable in many laboratories of developing countries. To circumvent this problem, biotinilated HPA, and alkaline phosphatase- or ferritin-conjugated HPA could be employed. (2) Only seven different bacterial genera—including 15 streptococcal and 3 staphylococcal strains—, were tested. It should be noted, however, that the bacteria tested here include some of the microorganisms most frequently causing CAP and other severe diseases. (3) Although pneumococci and staphylococci are morphologically different, the finding that two *S. aureus* strains also bind HPA may sometimes represent a diagnostic drawback and deserves further research. It should be noted, however, that, for example, severe sepsis—a serious complication of CAP—is caused by *S. pneumoniae* about 100 times more frequently than by *S. aureus*, whereas methicillin-resistant *S. aureus* is an important cause of antimicrobial-resistant hospital-acquired infections worldwide and remains a public health priority in Europe (Montull et al., 2016). (4) This represents an *in vitro* study and an appropriate evaluation of the benefits of HPA labeling for diagnostic purposes should be performed directly with clinical samples, e.g., sputum, brochoalveolar fluid, blood, and/or CSF.

## Author contributions

MD and EG conceived and designed the experiments. MD performed the experiments. MD and EG analyzed the data and wrote the paper.

### Conflict of interest statement

The other authors declare that the research was conducted in the absence of any commercial or financial relationships that could be construed as a potential conflict of interest. The reviewer ME declared a shared affiliation, though no other collaboration, with one of the authors MD to the handling Editor, who ensured that the process nevertheless met the standards of a fair and objective review.
